# Heatwaves enable wildfire activity in the western United States

**DOI:** 10.1126/sciadv.aea1277

**Published:** 2026-06-17

**Authors:** Dmitri A. Kalashnikov, John T. Abatzoglou, Emily L. Williams, Cong Yin, Madhulika Gurazada, Mukesh Kumar, Ashwin P. Thomas, Precious E. Ebiendele

**Affiliations:** ^1^ Sierra Nevada Research Institute, University of California, Merced, Merced, CA, USA.; ^2^ School of Engineering, University of California, Merced, Merced, CA, USA.

## Abstract

While overall impacts of heatwaves have been extensively studied, the connection between heatwaves and wildfire activity remains relatively underexplored. We analyze links between heatwaves and both wildfire occurrence and growth across the western United States (WUS) and find that 42% of burned area during 2001–2024 occurred during and immediately following heatwaves. Heatwaves facilitate significant increases in daily burned area through meteorological and fuel flammability conditions that promote new ignitions and exacerbate ongoing fire activity, with effects persisting post-heatwave in most regions. In addition, heatwaves co-occur with increased cloud-to-ground lightning that can potentially increase ignitions. Last, we observe a 2.5-fold increase in burned area in WUS forests since 2001, with ~64% of this increase coinciding with heatwaves, but without corresponding increases in nonforests. The growing influence of heatwaves in shaping burned area in WUS forests has important implications for fire management and public health and can improve predictions of wildfire risk.

## Teaser

Heatwaves are associated with a disproportionate share of wildfire activity and trends in the western US in recent decades.

## INTRODUCTION

Communities, ecosystems, and infrastructure across the western United States (WUS) face escalating threats from large and destructive wildfires. While many WUS ecosystems are fire-adapted, a convergence of contributing factors—including dry fuels, strong winds, “dry” lightning, and the expanding wildland-urban interface—make the WUS one of the most susceptible regions to high-impact fires worldwide (*1*). Recent decades have seen a marked increase in fire activity across the WUS, driven by warmer and drier conditions (*2*–*4*) as well as other factors such as increased fuel loads in some ecosystems stemming from, for example, historical fire suppression in dry forests (*5*, *6*) and the spread of invasive annual grasses in places such as the Great Basin (*7*, *8*). From 2000 to 2019, the WUS accounted for 82% of total US population exposure to large wildfires (*9*), and recent fire seasons have brought devastating consequences for life, property, and air and water quality (*10*–*14*).

Climatic influences on fire activity have been the subject of extensive research. Studies have shown that elevated seasonal temperatures and fuel dryness are strongly associated with interannual variability in burned area in flammability-limited, typically forest, ecosystems (*2*, *15*–*18*), while prior-year moisture and resultant buildup of fine fuels are recognized drivers of interannual burned area in fuel-limited, typically shrub- and grass-dominated, ecosystems (*15*, *19*–*21*). Similarly, the influence of meteorological extremes—such as strong winds and overall fire weather conditions (e.g., as quantified through the Fire Weather Index)—are well understood in the context of short-term fire risk (*22*, *23*). However, the influences of other short-term meteorological drivers of fire activity, such as heatwaves, have received less attention. While heatwaves are well-recognized threats to human health (*24*), energy supply (*25*), and ecosystems (*26*), emerging research suggests that they may also amplify fire activity (*27*–*32*). Heatwaves can rapidly dry fine fuels (*33*), limit overnight humidity recovery and foster longer burn periods (*34*), and increase atmospheric instability that promotes plume-dominated fire behavior and rapid fire growth (*35*–*37*). In addition, heatwaves may coincide with increased lightning activity, providing multiple ignition sources (*38*, *39*). However, heatwaves are not directly codified into operational fire weather warnings (e.g., Red Flag Warnings), which typically prioritize wind and humidity thresholds, thus potentially creating a gap in fire early warning systems.

**Fig. 1.**
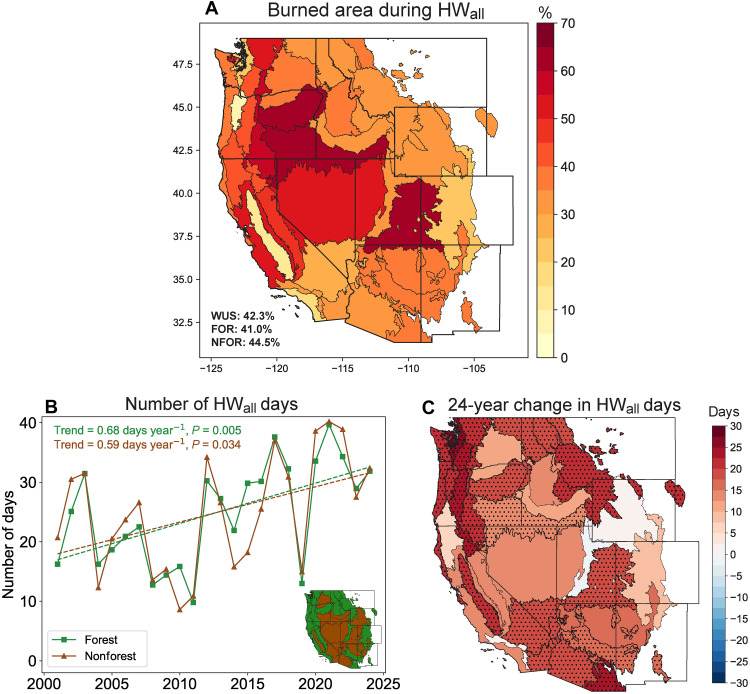
Association of heatwaves and burned area and changes in heatwave days. (**A**) Percent of total burned area that occurred during and 1 to 5 days after heatwaves (HW_all_) over May to October, 2001–2024. Inset text shows aggregate percentages over the WUS, forest ecoregions (FOR), and nonforest ecoregions (NFOR), respectively. (**B**) Number of HW_all_ days averaged over forest and nonforest ecoregions during May to October, 2001–2024. Significance of linear trends is determined using a two-tailed *t* test. Inset map shows EPA Level III ecoregions of the WUS classified as forest/nonforest (see Materials and Methods). See fig. S1 for ecoregion names. (**C**) As in (B), but with linear trends calculated by ecoregion and multiplied by the number of years (*n* = 24). Stippling indicates significant trends (*P* < 0.10) according to a two-tailed *t* test.

In this study, we address two key questions to improve our understanding of the relationship between heatwaves and fire activity across the WUS: (i) How do heatwaves affect fire activity, and how does this relationship vary across this region? And (ii), how have heatwaves, burned area, and their concurrence changed in recent decades? We integrate satellite-derived burned area, fire occurrence reports from federal and state agencies, and gridded meteorological variables during 2001–2024 and conduct our analyses separately for predominantly forested and nonforested ecoregions across the WUS (Fig. 1). As heatwaves are increasing in frequency and intensity in a warming climate, understanding their role in driving fire activity is critical for assessing future changes to fire regimes and informing management and adaptation strategies.

## RESULTS

### Seasonal heatwave-burned area relationships and trends

Using an ecoregion-level definition of heatwaves across the WUS (fig. S1), we find that ~42% of May–October (hereafter, “warm season”) burned area from 2001 to 2024 occurred either during or in the 5 days following heatwaves (“HW_all_” periods; see Materials and Methods) (Fig. 1A). This is particularly notable given that HW_all_ account for only ~12 to 15% of warm season days, depending on the ecoregion (fig. S2), indicating that heatwaves disproportionately shape daily fire activity in the WUS. Nonforest areas of the Intermountain West, particularly northern Nevada, southern Idaho, and eastern Oregon, show the highest proportion (>60%) of warm season burned area occurring during HW_all_ (Fig. 1A). The number of HW_all_ days increased by 91% in forest ecoregions (17 to 32.6 days year^−1^; *P* = 0.005) and by 76% in nonforest ecoregions (18 to 31.6 days year^−1^; *P* = 0.034) during 2001–2024 (Fig. 1B). Most of the ecoregions show significant increases in the number of HW_all_ days (Fig. 1C). In addition, the length of the heatwave season, defined as the number of days between the first and last heatwave day, expanded by 3 to 5 weeks across the WUS (fig. S3).

**Fig. 2.**
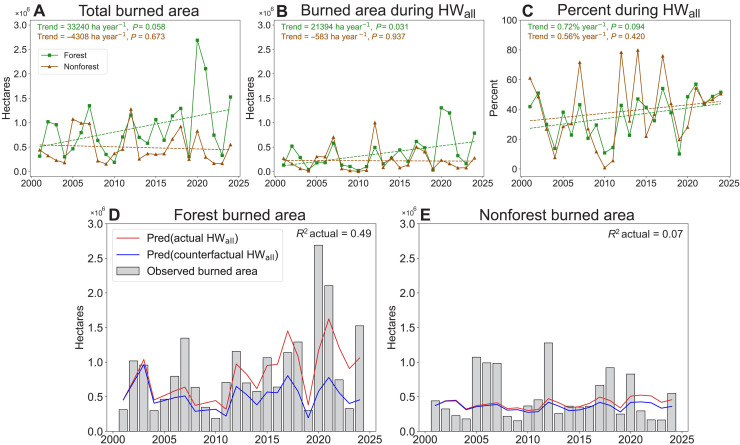
Changes in burned area and the role of heatwaves. Time series of (**A**) total burned area, (**B**) burned area that occurred during HW_all_, and (**C**) percent of total burned area that occurred during HW_all_, aggregated over forest and nonforest ecoregions. Significance of linear trends is determined using a two-tailed *t* test. (**D**) Linear regression results showing annual forest burned area predicted by the number of observed HW_all_ days (red line) and detrended HW_all_ days (counterfactual, blue line). Observed forest burned area shown by gray bars. (**E**) As in (D), but for nonforest burned area. *R*^2^, coefficient of determination.

Forest burned area increased significantly by ~2.5-fold (33,240 ha year^−1^; *P* = 0.058) during the study period with 2020, 2021, and 2024 being the top 3 years of forest fire extent (Fig. 2A). Meanwhile, forest burned area during HW_all_ has increased significantly by fivefold (21,394 ha year^−1^; *P* = 0.031)—accounting for 64% of the total increase in forest burned area (Fig. 2B). Correspondingly, a growing share of forest burned area has been associated with HW_all_, increasing from ~27 to ~44% since 2001 (*P* = 0.094; Fig. 2C). Forest ecoregions with significant overall burned area increases geographically overlap with those experiencing significant burned area increases during HW_all_ (fig. S4, A and B). Furthermore, even after normalizing burned area during HW_all_ by the number of HW_all_ days, there is a nonsignificant upward trend in daily burned area on HW_all_ days in forests (389.53 ha day^−1^ year^−1^; *P* = 0.16; fig. S5), representing a doubling of the burned area per HW_all_ day since 2001. This suggests that increased forest burned area during HW_all_ is not solely driven by increased HW_all_ days but likely reflects amplification of burning conditions by more frequent—and hotter—heatwaves that more effectively dry larger fuels (Fig. 1B) (*40*, *41*).

Conversely, nonforest burned area, including that occurring during HW_all_, shows nonsignificant decreases over the study period despite increased HW_all_ frequency (Fig. 2, A and B), likely due to the fuel-limited nature of these ecosystems. Despite the importance of heatwaves for daily-scale burned area in many nonforested regions of the WUS (Fig. 1A), high temperatures and rapid fuel drying are not prerequisites to large burned area on interannual timescales compared to prior-year moisture that promotes fine-fuel growth and enables fuel continuity (*15*, *20*, *21*, *42*). With increasing heat extremes not providing a strong control on nonforest burned area trends, the observed nonsignificant decreases could be related to increasing aridity that inhibits fine-fuel growth or confounding anthropogenic factors such as reduced human-caused ignitions during critical burning periods (*16*, *21*, *43*, *44*).

To further understand the interannual variation between heatwaves and burned area in forest and nonforest ecoregions, we develop linear regression models that use the number of warm-season HW_all_ days to predict season-total burned area. Notably, these simple univariate models explain 49% of the interannual variance in forest burned area but only 7% for nonforest burned area (Fig. 2, D and E). Despite the complexity of factors governing fire activity, these findings support the idea that the increased occurrence of heatwaves is an important contributor to increasing burned area in forests but has not meaningfully contributed to burned area trends in nonforest ecosystems. Furthermore, counterfactual regression models using detrended HW_all_ days (see Materials and Methods) additionally suggest that, absent the increase in HW_all_ days, cumulative forest burned area would have been ~37% lower over 2001–2024 (Fig. 2D).

We additionally note significant increases in synchronous heatwave conditions (defined as affecting at least half of all ecoregions in the forest or nonforest category), with an increase of 9.6 days year^−1^ (*P* = 0.018) across forest ecoregions and 8.6 days year^−1^ (*P* = 0.062) across nonforest ecoregions since 2001 (fig. S6). This suggests an expansion in the geographic coverage of heatwaves, posing potential challenges for fire management due to the linkage of heatwaves and fire activity (Fig. 1A). For example, the availability and mobility of fire suppression resources that are often shared across the WUS can become constrained during synchronous heatwaves, reducing the efficacy of response efforts and potentially amplifying burned area (*45*).

### Short-term impact of heatwaves on wildfire activity

**Fig. 3.**
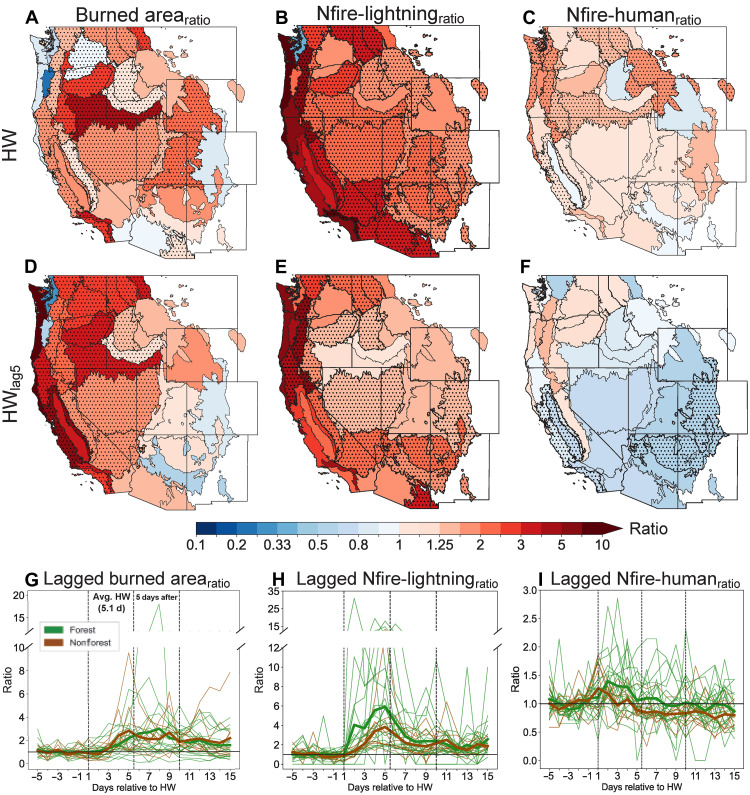
Ratios of burned area and fire counts compared to days preceding heatwaves. (**A**) Ratios of daily burned area during heatwaves (HW) compared to the 5 days preceding HW over May to October, 2001–2024, averaged from all HW in the period of record. (**B**) As in (A), but for the number of fires (Nfire) attributed to lightning from FPA-FOD over May to October, 2001–2020. (**C**) As in (B), but for human-caused fires. (**D** to **F**) As in (A) to (C), but comparing 1 to 5 days after the end of HW (HW_lag5_) to the 5 days preceding HW. Stippling indicates that distributions are significantly different (*P* < 0.10) according to a Kolmogorov-Smirnov test. (**G**) Ratios between daily burned area and the 5-day average burned area preceding the start of HW in each ecoregion, averaged from all HW during the period of record. Ratios are calculated for each day between the preceding 5 days and the following 15 days relative to HW start (day 1). Thin lines show individual ecoregions, while thick lines show the average of ecoregions in that land cover class. (**H** and **I**) As in (G), but for lightning- and human-caused fires, respectively.

Next, we examine the short-term impact of heatwaves on fire activity during and in the 5 days following heatwaves relative to preheatwave baseline conditions (defined as the average of the 5 days before the commencement of the heatwave; see Materials and Methods). We show that burned area during heatwaves increases significantly by ~1.5 to 4-fold relative to baseline conditions across much of the WUS, with the highest increases in the northern Great Basin (Fig. 3A). This finding suggests that shrub- and herbaceous-dominated landscapes respond more quickly to heatwaves due to faster curing and the potential for rapid wind-driven fire spread in finer fuels, particularly from thunderstorm downdrafts (*33*). Conversely, forest ecoregions, where larger fuels typically require longer curing periods and fire spread rates are generally slower, see larger burned area increases in the post-heatwave windows (Fig. 3D). Aggregated burned area ratio in nonforest ecoregions peaks four days after heatwave onset, while forest ecoregions peak 7 days after heatwave onset (Fig. 3G).

Similar to burned area, ignitions also show strong spatial and temporal variation. Lightning-caused fire counts increase significantly both during and following heatwaves compared to baseline conditions in many ecoregions (Fig. 3, B and E), with ratios exceeding fivefold relative to baseline conditions in forest ecoregions of western Oregon, Washington, and California. We note that lightning-caused fire counts peak across both forest and nonforest areas 4 days after heatwave onset (Fig. 3H), demonstrating the ability of heatwaves to catalyze new fire activity. However, this ratio is larger when aggregated across forests (~5.2-fold) compared to nonforests (~3.1-fold), likely reflecting the greater availability and continuity of fuels in forested environments that can sustain an ignition (*46*, *47*). The continued elevated ratio of lightning-caused fire counts post-heatwave across both forests and nonforests could result from delayed detection of lightning-caused fires (i.e., “holdover fires”) (*48*, *49*) or thunderstorms triggered by a post-heatwave transition to an atmospheric pattern that promotes additional convection (i.e., “ridge breakdown”) (*50*–*52*).

In contrast, human-caused ignitions mainly increase during heatwaves in forest ecoregions of the Northwest (Fig. 3C), likely due to enhanced landscape flammability and human factors including increased outdoor recreation introducing new ignitions to the landscape during hot and dry periods (*53*). Meanwhile, the Southwest shows largely weak and nonsignificant changes in human-caused fire counts during heatwaves compared to baseline conditions (Fig. 3C). This may be due to heatwaves deterring outdoor recreation in hotter climates, thus limiting ignitions. None of the ecoregions show significant increases in human-caused fire counts in the 5 days post-heatwave (Fig. 3F). Conversely, several interior Southwest ecoregions show significant declines in human-caused fire counts post-heatwave, likely reflecting increased monsoonal moisture and precipitation coincident with the end of heatwave conditions (Fig. 3F).

### Influence of heatwaves on meteorological and fuel moisture characteristics

**Fig. 4.**
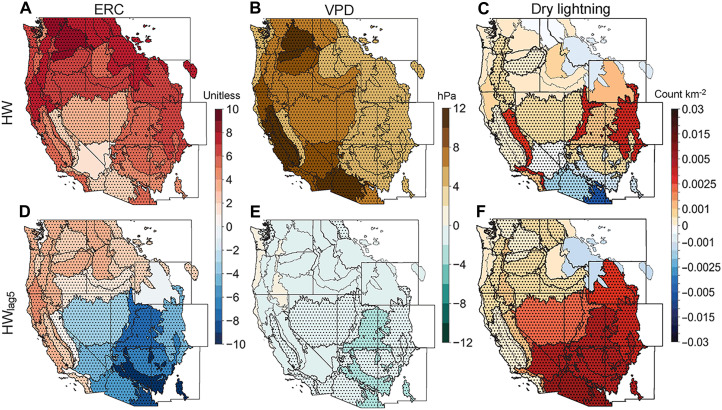
Differences in meteorological variables compared to days preceding heatwaves. Differences in (**A**) ERC, (**B**) VPD, and (**C**) number of daily dry lightning flashes per km^2^ between heatwaves (HW) and the 5 days preceding HW (during HW minus before HW) over May to October, 2001–2024, averaged from all HW in the period of record. Dry lightning is defined as the concurrence of cloud-to-ground lightning from the National Lightning Detection Network with <2.5-mm daily-accumulated precipitation from gridMET. (**D** to **F**) As in (A) to (C), but comparing 1 to 5 days after the end of HW (HW_lag5_) to the 5 days preceding HW (after HW minus before HW). Stippling indicates that distributions are significantly different (*P* < 0.10) according to a Kolmogorov-Smirnov test.

The energy release component (ERC) and vapor pressure deficit (VPD) are elevated during heatwaves across the study domain (Fig. 4, A and B). These results are expected outcomes of heatwaves realized through higher maximum temperatures (*T*_max_) that in turn drive lower daily-minimum relative humidity (*RH*_min_) and increased atmospheric water demand, thus rapidly drying and increasing the availability of fuel for combustion (fig. S7, A and C). Notably, ERC remains significantly elevated following heatwaves in the Northwest and central and northern California (Fig. 4D), indicating continued high fire danger and likely contributing to the elevated burned area post-heatwave (Fig. 3D). As ERC is heavily weighted toward 100- and 1000-hour dead fuel moisture (*54*), this moisture memory appears to be an important factor in the observed lagged effects of heatwaves on fire activity despite *T*_max_, *RH*_min_, and VPD returning to their preheatwave values (Fig. 4E and fig. S8, A, C, and F to G). By contrast, sharp post-heatwave declines in ERC and VPD are observed in the interior Southwest (Fig. 4, D and E) due to the resumption of monsoonal moisture and precipitation that simultaneously ends the heatwave and reduces fire danger (Fig. 3D and fig. S8, A and E) (*55*, *56*).

We also show that dry lightning activity (cloud-to-ground lightning occurring with <2.5-mm daily-accumulated precipitation) increases both during and after heatwaves relative to baseline conditions across the majority of the WUS (Fig. 4, C and F). During heatwaves, dry lightning increases relative to “wet” lightning (≥2.5-mm precipitation) in many ecoregions (Fig. 4C and fig. S9B). In many areas of the WUS away from the monsoon core, dry lightning is more likely during heatwaves as hotter, drier conditions in the lower troposphere increase the evaporation of rainfall before it reaches the ground (*38*, *57*). In the monsoon core of the Southwest, convection is less likely during heatwaves as they typically occur either before monsoon season onset or during breaks in monsoon activity. Overall, the concurrence of increased dry lightning and dry fuels associated with heatwaves, particularly outside the monsoon core, enhances the likelihood of lightning-caused ignitions and increased burned area (Fig. 3).

### Differential impacts of dry versus moist heatwaves

**Fig. 5.**
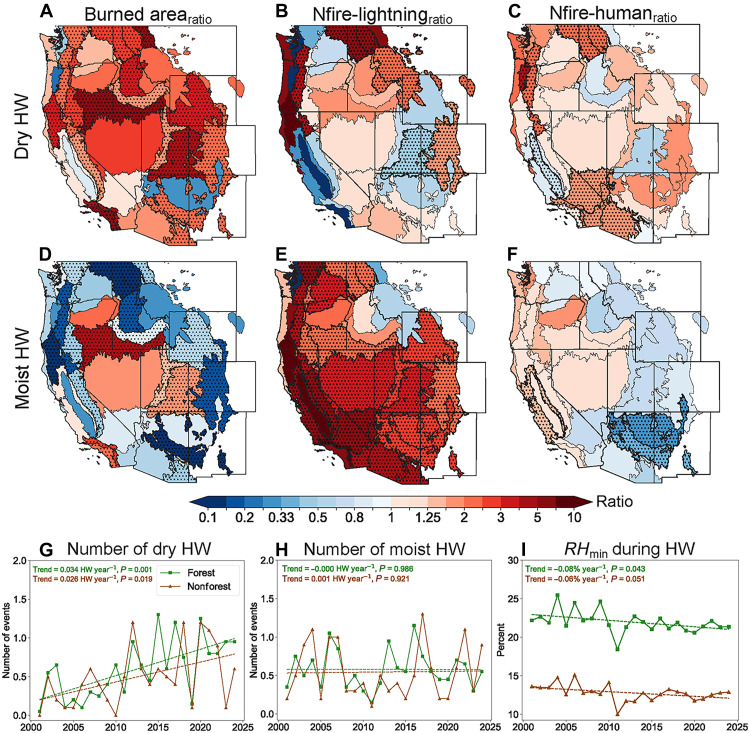
Differences between dry and moist heatwaves. Ratios as in Fig. 3 (A to C), but for (**A** to **C**) dry heatwaves (HW; average daily-minimum relative humidity (*RH*_min_) <25th percentile of the May to October distribution) and (**D** to **F**) moist HW (average *RH*_min_ > 75th percentile of the May to October distribution). Number of (**G**) unique dry HW events and (**H**) unique moist HW events averaged over forest (*n* = 20) and nonforest (*n* = 10) ecoregions. (**I**) *RH*_min_ during HW averaged over forest and nonforest ecoregions. Significance of linear trends in (G) to (I) is determined using a two-tailed *t* test.

Because of the importance of humidity in driving fire weather and subsequent wildfire potential (*18*), we hypothesize that humidity exerts a strong control on fire activity during heatwaves. To test this hypothesis, we compare the ratios of burned area and counts of lightning- and human-caused fires during both dry and “moist” heatwaves against baseline conditions (Fig. 5). Dry and moist heatwaves are defined using thresholds of *RH*_min_ averaged over the length of the heatwave (<25th and >75th percentiles; see Materials and Methods). During dry heatwaves, burned area is significantly higher than baseline conditions across most ecoregions (Fig. 5A). This effect generally persists in the post-heatwave period as well (fig. S10). In contrast, forest ecoregions during moist heatwaves typically experience reduced burned area compared to baseline conditions (Fig. 5D). Fire weather is significantly dampened during moist compared to dry heatwaves nearly everywhere (fig. S11). However, moist heatwaves still promote increased burned area in the Great Basin by up to fourfold compared to baseline conditions (Fig. 5D). In these dry ecoregions, the 75th percentile of *RH*_min_ is likely sufficiently low in an absolute sense to not meaningfully affect flammability, allowing for the increased lightning activity and ignitions during moist heatwaves to contribute to a significant increase in burned area (Fig. 5, D and E).

Notably, we observe a fivefold and fourfold increase in dry heatwave frequency since 2001 across forest and nonforest ecoregions, respectively (Fig. 5G). Conversely, moist heatwave frequencies do not exhibit any significant trends (Fig. 5H). Alongside the increasing frequency of dry heatwaves, we report significant declines in *RH*_min_ during heatwaves within both forest (−0.08% year^−1^, *P* = 0.043) and nonforest (−0.06% year^−1^, *P* = 0.051) ecoregions (Fig. 5I). We additionally show that declines in *RH*_min_ are most pronounced in forest ecoregions of Washington, Oregon, and California (fig. S12). These areas overlap with the largest increases in burned area, suggesting that progressively drier heatwaves have become more effective in amplifying fire risk (Fig. 2B and fig. S4A).

## DISCUSSION

Our results demonstrate that heatwaves both catalyze and enable warm-season wildfire activity across the WUS. While HW_all_ periods (consisting of both heatwaves and the 5 days immediately post-heatwave) represent only ~12 to 15% of warm-season days depending on the ecoregion, they accounted for ~42% of total burned area during May to October, 2001–2024 (Fig. 1A). Moreover, we show that daily burned area during heatwaves increases by more than 50% across a majority of WUS ecoregions relative to preheatwave conditions (Fig. 3A). This disproportionate contribution underscores how heatwaves fundamentally shape daily to interannual fire activity—not only by increasing fuel flammability via prolonged elevated fire weather conditions but also by extending the period of heightened fire risk well beyond the heatwave itself.

Forest burned area during HW_all_ has grown more than fivefold during 2001–2024, substantially outpacing the overall trend in warm-season forest burned area and accounting for 64% of this increase (Fig. 2, A and B). We also find that HW_all_ days explain nearly half of the interannual variability in forest burned area, and our counterfactual modeling indicates that cumulative forest burned area would have been 37% lower during 2001–2024 without the increase in HW_all_ days (Fig. 2D). Increased burned area in forests has the potential for outsized smoke impacts compared to nonforests due to the larger quantities of biomass available for burning (*58*). Our findings suggest that heatwaves are not only coincident with but at least partially drive a substantial portion of the escalation in WUS forest fire activity this century. Yet, the observed increase in forest burned area during heatwaves cannot be explained solely by the increased HW_all_ days (fig. S5). This suggests a nonlinear response that implicates the cumulative drying of heavier fuels due to more frequent heatwaves (Figs. 1, B and C, and 5I), persistent fuel aridity following heatwaves (Fig. 4D), larger and faster-growing fires enabled by drier atmospheric conditions (Fig. 5, G and I), and a reduced nighttime barrier to fire through elevated overnight temperatures and lower humidity (*34*, *59*), in addition to the potential for reduced response efficacy from increased concurrence of multiple large fires straining suppression resources (*45*). The increasing frequency of HW_all_ days and a longer heatwave season likely reflect both background warming and increased occurrence of high-pressure ridging over the WUS (*14*, *60*, *61*). These ridging episodes, through their ability to suppress precipitation and increase surface temperatures, have been linked to increased fire activity in the WUS (*37*). By highlighting the role of heatwaves, our results provide nuance to recent work implicating antecedent winter and spring climate as the dominant drivers of interannual variability and trends in WUS burned area (*62*) and align with recent findings on the outsized role of heatwaves driving boreal forest burned area across the Northern Hemisphere (*31*).

Nonforest ecosystems—often dominated by grasses and shrubs—exhibit high proportions of seasonal burned area occurring during HW_all_ periods, particularly in the Great Basin. More than 60% of seasonal burned area is linked to HW_all_ in these regions, highlighting their sensitivity to daily-scale temperature extremes (Figs. 1A and 3A). However, burned area trends in nonforests have remained flat or declined despite more frequent heatwaves (Fig. 2A). This discrepancy reflects the fuel-limited nature of nonforest systems, where aggregate burned area is more contingent on antecedent wet growing seasons that promote fine-fuel growth than on concurrent meteorological conditions (*19*–*21*, *54*).

Our results suggest several processes through which heatwaves serve as both a catalyst and enabler of fire activity—preconditioning the landscape for combustion, exacerbating ongoing fire activity, and increasing dry lightning. First, prolonged anomalously hot temperatures during heatwaves desiccate fuels and increase landscape flammability (e.g., increased ERC and VPD), thereby facilitating increased fire potential where fuels are available (Fig. 4, A and B). In addition, the diurnal cycle can become more conducive to fire during heatwaves—hot afternoon temperatures provide direct thermal energy to fuels, thereby raising their temperature closer to the point of ignition, while calm winds under a warm airmass promote the formation of nighttime thermal belts with persistently warm overnight temperatures on lower- to mid-elevation mountain slopes limiting nocturnal recovery of VPD and favoring longer burning periods, including overnight burning (*34*, *63*). Second, longer-lived flammability metrics (e.g., ERC) in forested areas of the WUS not influenced by the monsoon remain elevated after the termination of heatwaves (Fig. 4D), enabling prolonged burning periods and sustained nonlinear growth of active fire lines that increase burned area in the days following heatwaves (Fig. 3D) (*64*). Last, in addition to desiccating fuels, we show that heatwaves are associated with increased lightning activity, including dry lightning (Fig. 4C) that can ignite vegetation in the absence of sufficient wetting rainfall (*49*, *57*). This is likely driven by increased thunderstorms, particularly over elevated terrain where orographic lifting is strongest, as the same high-pressure ridges that produce the heatwaves can induce lower- and mid-tropospheric instability and northward advection of monsoonal moisture (*38*, *51*). These effects are further compounded by human-caused ignitions, which increase across most of the Northwest during heatwaves, likely due to exposure of landscapes to accidental or negligent ignitions during high-risk periods (*53*, *65*).

Despite these findings, we also demonstrate that not all heatwaves are equally conducive to fire activity. We find that dry heatwaves are far more effective at increasing fire activity in forest ecoregions compared to moist heatwaves (Fig. 5, A and D). These findings have important implications for future projections of heatwave-enabled fire activity, particularly since observed drying humidity trends in the WUS are not well captured by climate models (*66*). We show that dry heatwaves have become more frequent across both forest and nonforest ecoregions, increasing fivefold and fourfold, respectively, since 2001 (Fig. 5G), complementing prior studies that reported drying trends during heatwaves in the Southwest (*67*). Concurrently, we show that daily-minimum relative humidity has declined significantly during heatwaves (Fig. 5I), especially in forest ecoregions of California, Oregon, and Washington—areas that also exhibit the steepest increases in burned area.

This study is subject to several potential limitations stemming from the scale of analysis, methodological choices, and data sources. First, the use of ecoregion-averaged heatwave definitions may obscure sub-ecoregion variability in temperature extremes, as localized areas may not experience heatwaves even when regional thresholds are exceeded and vice versa. While we separate heatwaves into dry and moist varieties, future work could disentangle the role of other heatwave characteristics such as length and intensity in modulating fire activity across the WUS. Second, our definition of heatwaves focuses on ≥3-day events and does not capture shorter (1 to 2 days) hot periods, which may also meaningfully influence fire activity. Third, we do not account for the frequency or clustering of heatwaves within a given fire season, which could affect cumulative fuel drying and fire activity. Last, although the Moderate Resolution Imaging Spectroradiometer (MODIS) provides a consistent and widely used satellite-based daily burned area dataset, these data are subject to uncertainties in detection stemming from cloud cover or dense wildfire smoke during satellite overpasses (*68*). Moreover, while most modern burned area in the WUS results from wildfires, some may originate from other sources including intentional agricultural fires and fire management activities (e.g., prescribed and cultural burns and backburning operations during active fires).

Nonetheless, our findings have clear implications for wildfire risk modeling and operational preparedness, and they are broadly applicable to regions globally where heatwaves drive fire activity (*30*–*32*). Current fire danger rating systems and early warning frameworks (e.g., Red Flag Warnings) do not explicitly account for heatwaves as a fire-enhancing mechanism. While these systems are implemented at finer spatial and temporal scales than our ecoregion-level analysis, the evidence presented here suggests that heatwaves—especially those accompanied by low humidity—consistently precondition fuels, coincide with increased lightning ignitions, and enable increased fire activity in many areas of the WUS. Our findings provide a broader perspective to those of Gutierrez *et al.* (*27*), who reported significant increases in burned area and fire occurrence in California’s Sierra Nevada ecoregion on hotter days. Likewise, our findings complement empirical modeling efforts for burned area that use seasonally averaged climate to understand past and future fire activity (*2*, *4*). Our results suggest that heatwave diagnostics could complement operational fire management and future wildfire projections, thereby offering the potential to mitigate the escalating impacts of wildfires on communities, ecosystems, and infrastructure across the WUS. As climate change continues to increase the frequency, duration, and intensity of heatwaves and the frequency of co-occurring drought and heatwave events, the window of opportunity for large and extreme fires will likely expand further absent wholesale changes in fuels (*69*–*71*). Moreover, the increasing spatial synchrony of heatwaves across multiple ecoregions poses logistical challenges for resource allocation. With more areas experiencing simultaneous high fire danger, firefighting personnel and suppression resources that are typically shared on regional to international scales can become stretched thin, reducing response effectiveness (*45*, *72*, *73*). We conclude that heatwaves are an amplifier of wildfire risk across the WUS and increasingly so in forests where they drive a majority of recent burned area increases. Their increasing frequency and intensifying aridity have important implications for fire regimes across the region, necessitating informed management and adaptation strategies.

## MATERIALS AND METHODS

### Study domain and season

We conduct our analyses over the conterminous WUS during the May to October warm season, which corresponds to the summer fire season responsible for the vast majority of WUS ignitions and burned area (*62*, *64*). We use the United States Environmental Protection Agency (EPA) Level III ecoregions as the unit of analysis (www.epa.gov/eco-research/level-iii-and-iv-ecoregions-continental-united-states), which are regions of broadly similar ecology and natural resource management needs (*74*). We include the 30 ecoregions that encompass WUS biomes from the Rocky Mountains westward (fig. S1). The EPA ecoregions are a hierarchical spatial framework with multiple levels of aggregation, ranging from coarse (level I) to fine (level IV) (*74*). Leveraging the information contained in their level I/II/III names, we further classify the ecoregions as forest or nonforest, with the forest classification including woodlands. To start, 24 of the 30 ecoregions contain “forest” or “desert” in their level I names and were assigned accordingly. Of the remaining six ecoregions, five contain either “mountains,” “highlands,” or “woodland” in either of their level I/II/III names and were thus assigned to the forest class. The remaining ecoregion (“Central California Valley”), although lacking land cover information in the metadata, was classified as nonforest due to the prevalence of agricultural land in this region. A total of 20 ecoregions are classified as forest, and 10 are classified as nonforest (Fig. 1B, inset). All datasets used in this study are aggregated to ecoregions before analysis. Statistical significance of trends is determined using a two-tailed *t* test (*P* < 0.10).

### Fire data

This study uses the daily burned area product MCD64A1 Collection 6 from MODIS at 500-m spatial resolution from 2001 to 2024 (*68*). Although this product captures burned area from all sources including agricultural burning, most detected burned area during the WUS summer season is presumed to be wildfire-related. We note that a sensor outage onboard the Terra MODIS satellite in June 2001 resulted in largely missing burned area observations between 15 June and 3 July that year (*75*). However, this was a below-average period for wildfire activity in the WUS, thus suggesting minimal influence on our results. Substituting daily MODIS burned area with estimates from the Fire Program Analysis Fire-Occurrence Database [FPA-FOD; (*76*)] over this period does not meaningfully alter our results (e.g., the total WUS percentage of burned area during HW_all_ is within ~0.02% of the raw data). The substitution was done by assigning final fire sizes from FPA-FOD as daily burned area on their ignition dates, likely overestimating their contribution on a given day as larger fires can burn over periods of days to weeks. Fire count data (>1 acre) are sourced from the FPA-FOD that aggregates all fires from reporting agencies in the United States between 1992 and 2020. We use FPA-FOD data over 2001–2020 to ensure a common start period with the MODIS burned area data. We separately analyze natural and human-caused fires, with natural fires presumed to be lightning-caused and referred to as such throughout the manuscript (*49*). Particularly for lightning-caused fires, we note that fire occurrences do not necessarily reflect the true number of ignitions and are thus a conservative estimate, as multiple proximate ignitions can merge into a single fire represented by one database entry in FPA-FOD (*77*).

### Meteorological and fuel moisture variables

To investigate the mechanisms linking heatwaves to fire activity in the WUS, we examine changes in several proxy variables between heatwaves and post-heatwave periods compared to the preheatwave baseline conditions. Gridded meteorological and fuel moisture variables, including all derived quantities, are from gridMET at 4-km spatial resolution (*78*). These variables include ERC from the US National Fire Danger Rating System and VPD, both representing widely used proxies for fuel aridity and fire potential in fire-climate studies (*17*, *34*, *54*). We consider other variables as well (see table S1 for full list of variable names and abbreviations). We also examine heatwave-related changes in dry lightning due to its outsized role in WUS summertime fire activity (*20*, *39*, *49*). Cloud-to-ground lightning data are from the National Lightning Detection Network (NLDN; 0.1° × 0.1°) and obtained from the National Centers for Environmental Information Severe Weather Data Inventory (www.ncei.noaa.gov/pub/data/swdi/database-csv/v2/). To identify dry and wet lightning, NLDN lightning data are matched to gridMET daily-accumulated precipitation that was bilinearly interpolated to the NLDN 0.1° grid. Dry lightning is defined as the concurrence of cloud-to-ground lightning with <2.5-mm daily-accumulated precipitation following previous work (*38*, *57*), while cloud-to-ground lightning occurrence with daily-accumulated precipitation ≥2.5 mm is termed wet lightning.

### Heatwave definition

Heatwaves are defined within each ecoregion as the occurrence of at least three consecutive days with ecoregion-average *T*_max_ > 90th percentile of the warm-season distribution, calculated over the full 2001–2024 period. *T*_max_ values representing the 90th percentile range from 25.7°C in the Rocky Mountains of western Montana to 41.9°C in the Sonoran Desert of southern Arizona and California (fig. S13). We note that the average heatwave length is 5.1 days among all ecoregions over the WUS (Fig. 3G). To examine potential lagged effects of heatwaves on fires, we also consider the 5 days that immediately follow the end of heatwaves (“HW_lag5_”), exclusive of any subsequent heatwaves in that window. To capture all days that might be influenced by heatwaves, we also analyze the combined window that includes both heatwaves and HW_lag5_ (HW_all_).

Since heatwaves can occur in conjunction with a variety of atmospheric moisture and evaporative demand conditions, we additionally analyze dry and moist heatwaves separately to examine their potentially differing influence on fire activity. Dry and moist heatwaves are defined based on daily *RH*_min_ averaged over the length of the heatwave event. A heatwave is termed dry (moist) if daily-average *RH*_min_ is <25th percentile (>75th percentile) of the warm-season distribution, calculated over 2001–2024. We note that *RH*_min_ > 75th percentile may still represent relatively dry conditions in an absolute sense over arid regions, making this a relative definition.

### Quantifying effects of heatwaves on wildfire activity

We quantify the effects of heatwaves on fire activity using three distinct analyses. First, we compare burned area summed from HW_all_ days to burned area summed from non-HW_all_ days over each warm season and over the period of record (Fig. 1). Next, we construct univariate linear regression models to predict the base-10 logarithm of season-total burned area (BA) over forest and nonforest ecoregions, respectively (Fig. 2). These models use only the season-total number of HW_all_ days, averaged over all forest and nonforest ecoregions, as predictors

$${\text{log}}_{10}\left({\text{BA}}_{\text{forest}}\right)=\beta_{0}+\beta_{1}{\text{HW}}_{\text{all},\text{forest}}$$

$${\text{log}}_{10}\left({\text{BA}}_{\text{nonforest}}\right)=\beta_{0}+\beta_{1}{\text{HW}}_{\text{all},\text{nonforest}}$$

We also construct counterfactual regression models that use the detrended number of HW_all_ days averaged over all forest and nonforest ecoregions as the predictor variable, obtained by removing the linear slopes in the frequency of HW_all_ days over 2001–2024. The counterfactual models capture the amount of burned area that would have occurred in each year without the observed increases in HW_all_ days.

To further disentangle the effects of heatwaves on fire activity, we divide daily-average burned area during heatwaves, HW_lag5_, and HW_all_ by the daily-average baseline burned area during all five-day preheatwave periods, exclusive of any other preceding heatwaves in that window, and repeat the procedure for fire counts (lightning- and human-caused) (Fig. 3). Calculating ratios in this manner controls for the concurrence of seasonal maxima in both heatwaves and fire activity during July–August across most of the WUS (*79*), as heatwaves inherently capture elevated fire activity compared to nonheatwave days that occur chiefly during May to June and September to October. Likewise, this approach allows us to compare the daily evolution of burned area and fire occurrence to the pre-heatwave control baseline. Values of meteorological and fuel moisture variables during heatwaves and HW_lag5_ are compared to preheatwave values by calculating arithmetic differences between their daily averages. Differences in daily distributions of burned area, fire counts, and meteorological and fuel moisture variables between heatwaves, HW_lag5_, and preheatwave periods are assessed for significance using the Kolmogorov-Smirnov test (*P* < 0.10).
