# Supplementary material for: Heatwaves enable wildfire activity in the western United States
Source: Sci Adv. 2026 Jun 17;12(25):eaea1277. doi: 10.1126/sciadv.aea1277 (PMC13274587; doi:10.1126/sciadv.aea1277)
Supplement: Supplementary file 1 — Table S1 Figs. S1 to S13 [file sciadv.aea1277_sm.pdf]

Science Advances
AAAS

Supplementary Materials for

Heatwaves enable wildfire activity in the western United States

Dmitri A. Kalashnikov *et al.*

Corresponding author: Dmitri A. Kalashnikov, dkalashnikov@ucmerced.edu

Sci. Adv. **12**, eaea1277 (2026)
DOI: 10.1126/sciadv.aea1277

This PDF file includes:

Table S1
Figs. S1 to S13

Variable Name	Abbreviation
Energy Release Component	ERC
Vapor Pressure Deficit	VPD
Maximum temperature	T_{max}
Fire Weather Index	FWI
Daily-minimum relative humidity	RH_{min}
Daily-average wind speed	Wind Speed
Daily-total precipitation	Precipitation
100-hour fuel moisture	FM_{100}
1000-hour fuel moisture	FM_{1000}

Table S1. Variables used and their abbreviation.

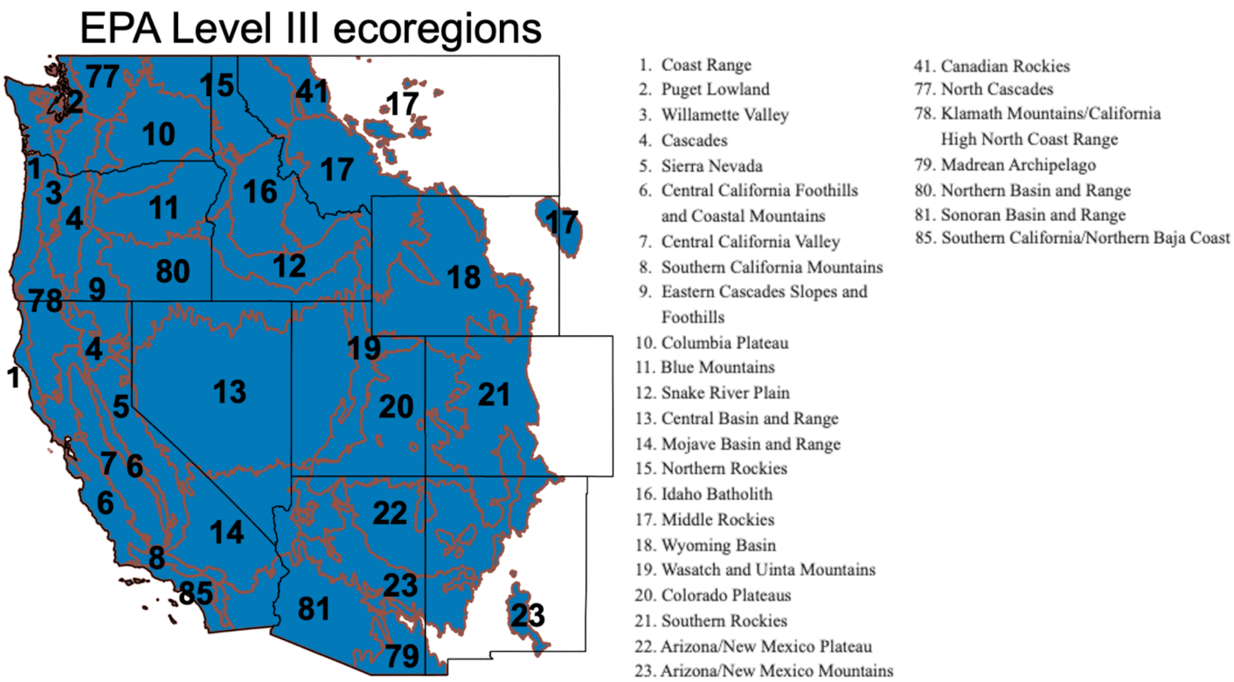

Fig. S1. EPA Level III ecoregions and names.

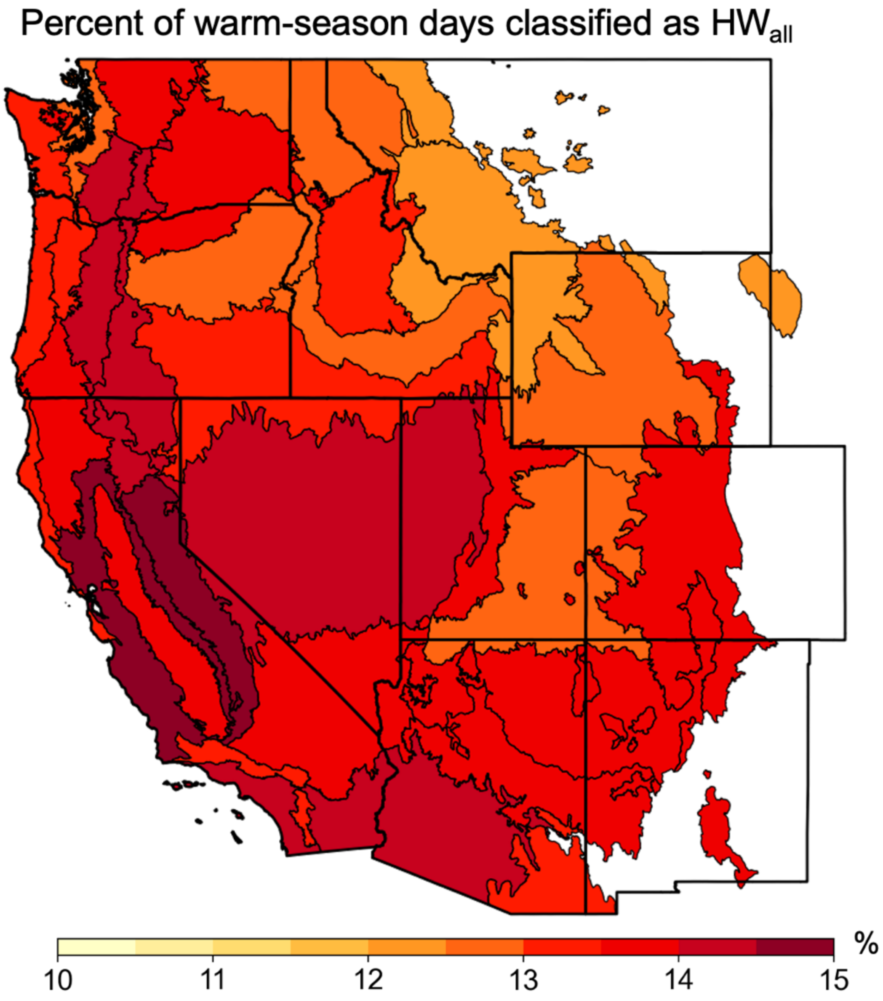

Fig. S2. Percent of warm-season days that are classified as either heatwaves or the 5 days following heatwaves (“HW_{all}”) during May-October, 2001-2024.

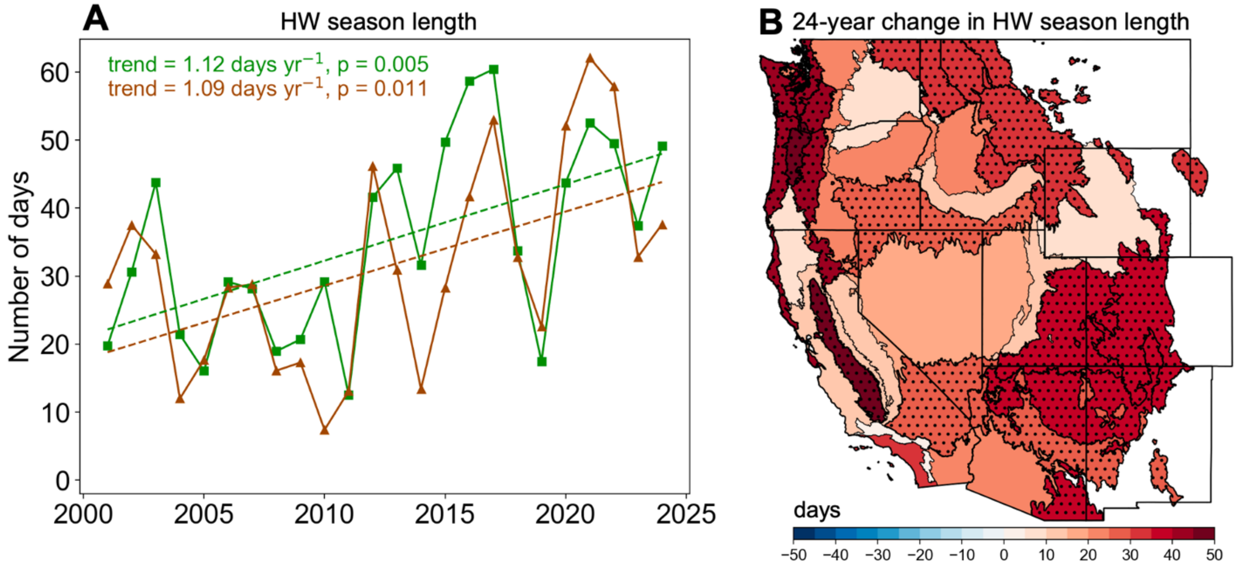

Fig. S3. (A) Heatwave season length, calculated as the difference between the first and last heatwave-classified Julian day. Significance of linear trends is determined using a two-tailed *t*-test. (B) As in (A), but with linear trends calculated by ecoregion and multiplied by the number of years ($n = 24$). Stippling indicates significant trends ($p < 0.10$) according to a two-tailed *t*-test.

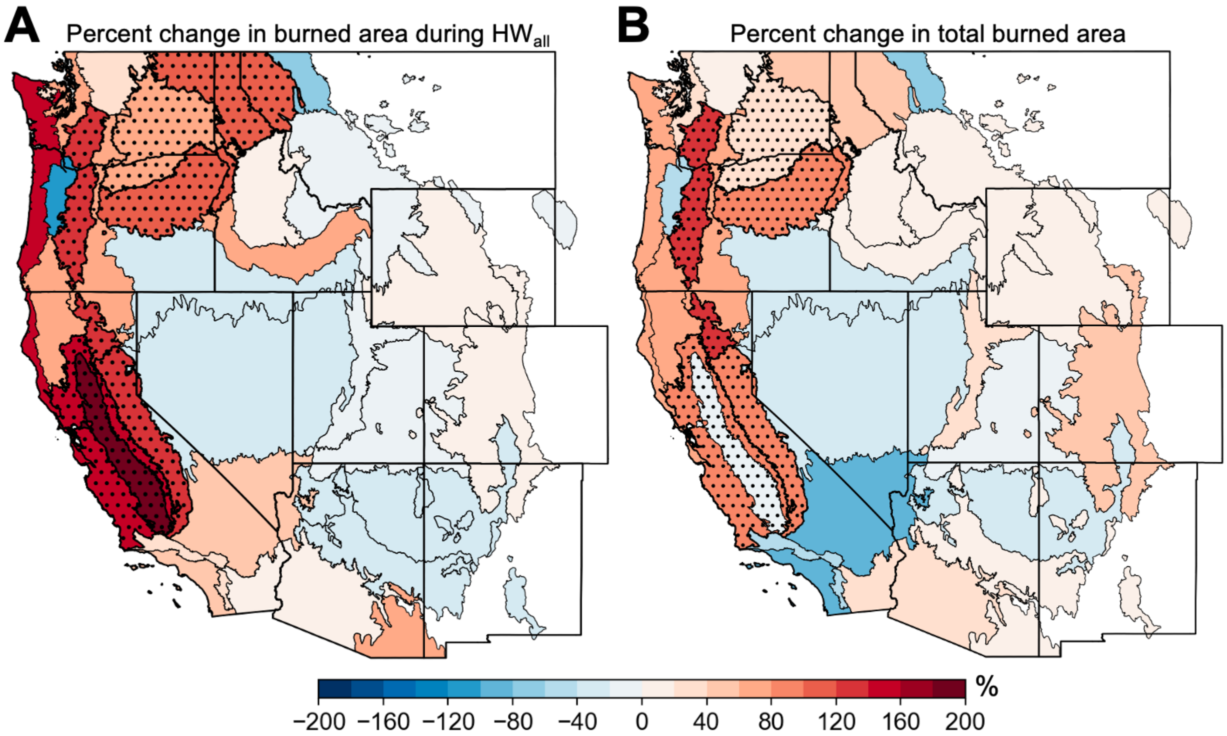

Fig. S4. (A) Percent change in burned area by ecoregion during HW_{all} and (B) on all days during May-October, 2001-2024. Stippling indicates significant trends ($p < 0.10$) according to a two-tailed *t*-test.

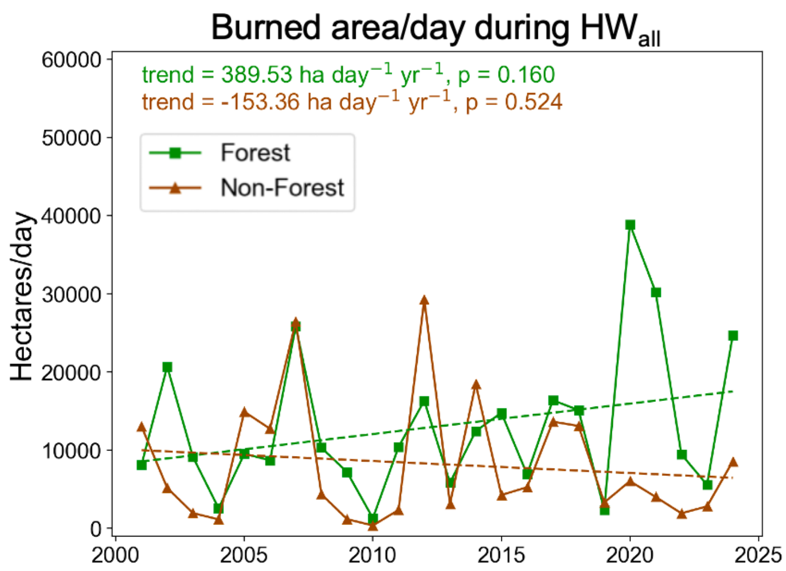

Fig. S5. As in Fig. 2A, but for burned area per day during HW_{all}. Significance of linear trends is determined using a two-tailed *t*-test.

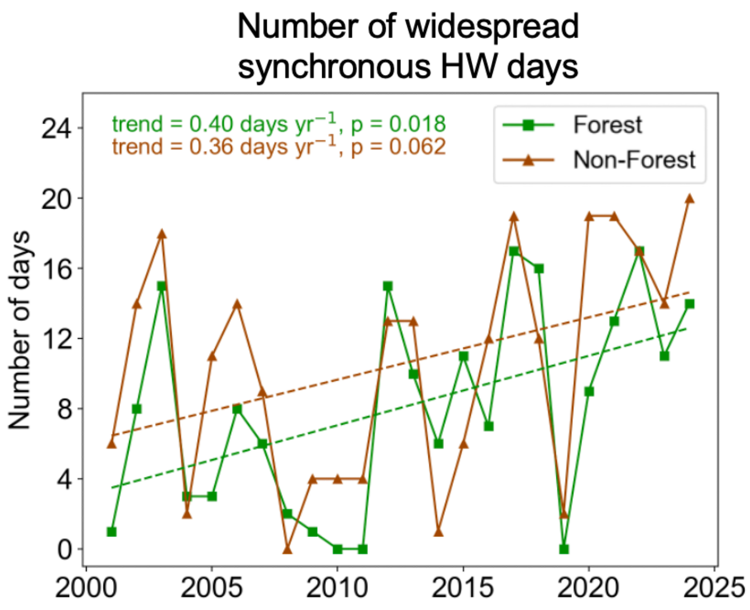

Fig. S6. Number of days when at least half of all forest or non-forest ecoregions experienced simultaneous heatwave days ("widespread synchronous HW days"). Significance of linear trends is determined using a two-tailed *t*-test.

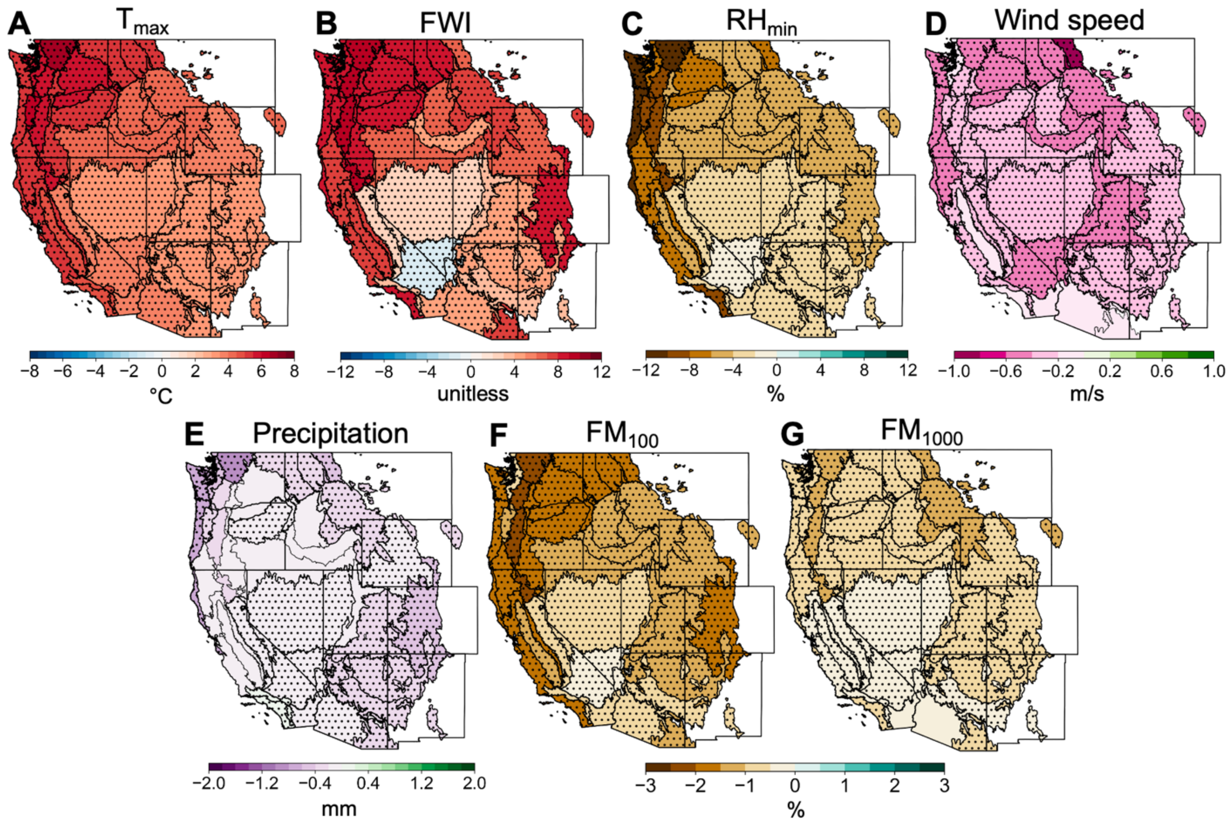

Fig. S7. Differences between heatwaves and the 5 days preceding heatwaves as in Fig. 4A-C, but for other meteorological and fuel moisture variables relevant to fire activity.

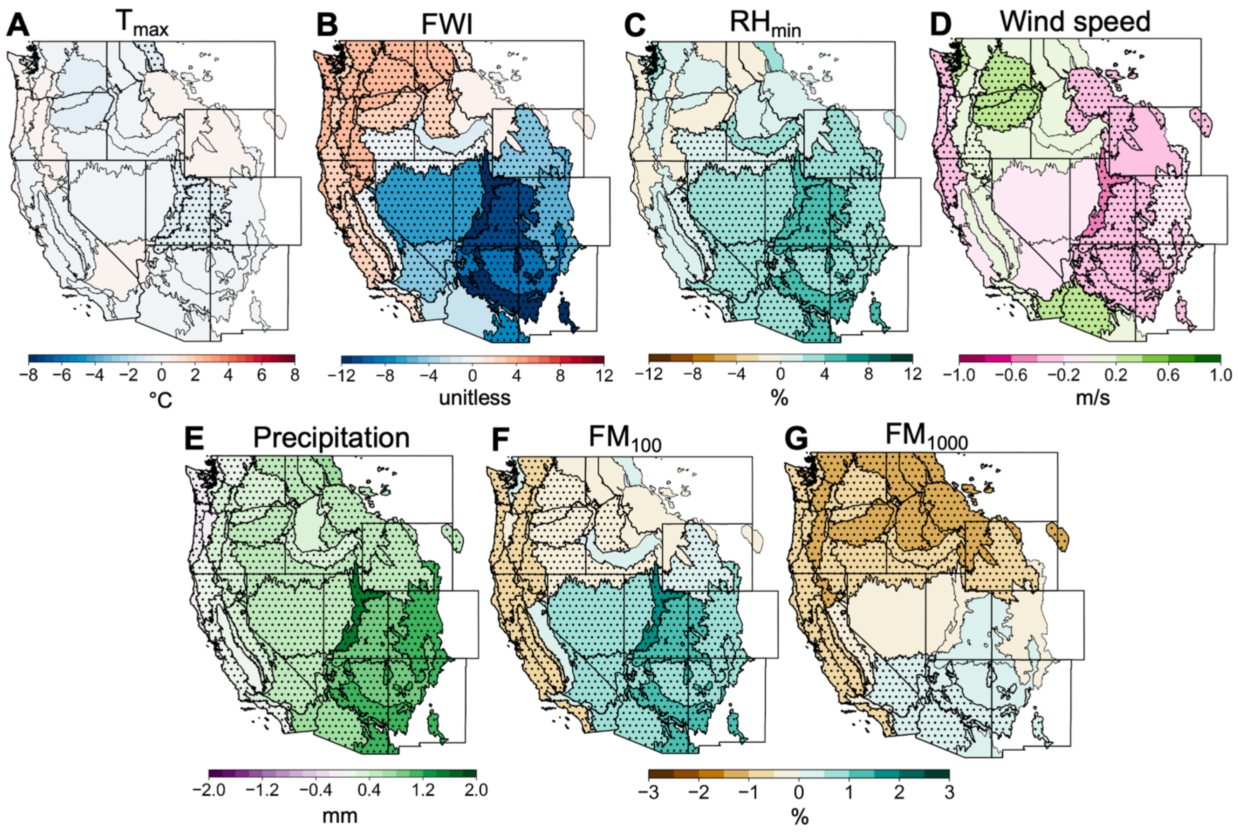

Fig. S8. As in Fig. S7, but comparing 1-5 days after the end of heatwaves (HW_{lag5}) to the 5 days preceding heatwaves.

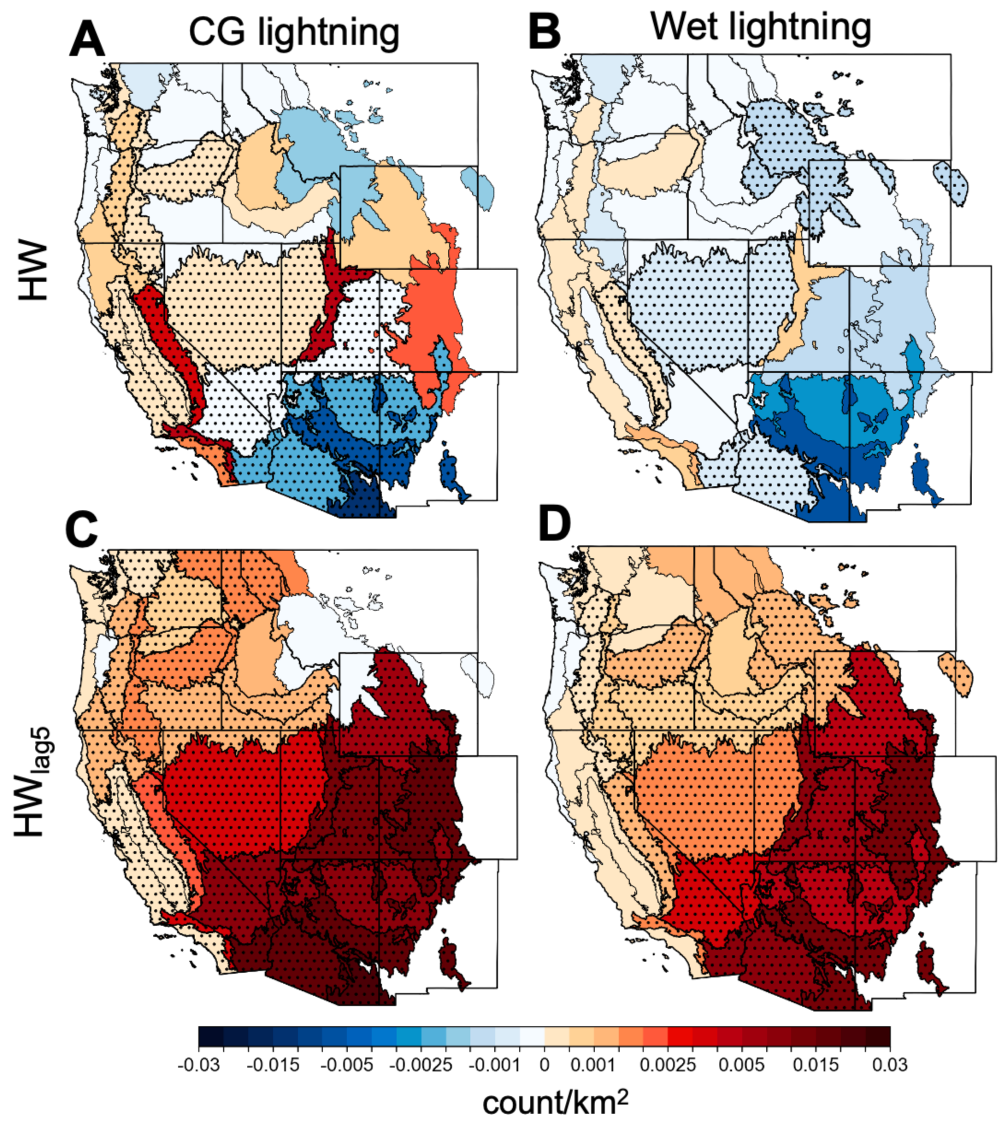

Fig. S9. As in Fig. 4, but for (A,C) total cloud-to-ground (CG) lightning and (B,D) "wet" lightning. Wet lightning is defined as the concurrence of CG lightning with ≥2.5 mm daily-accumulated precipitation. Total CG lightning is the sum of wet and dry lightning.

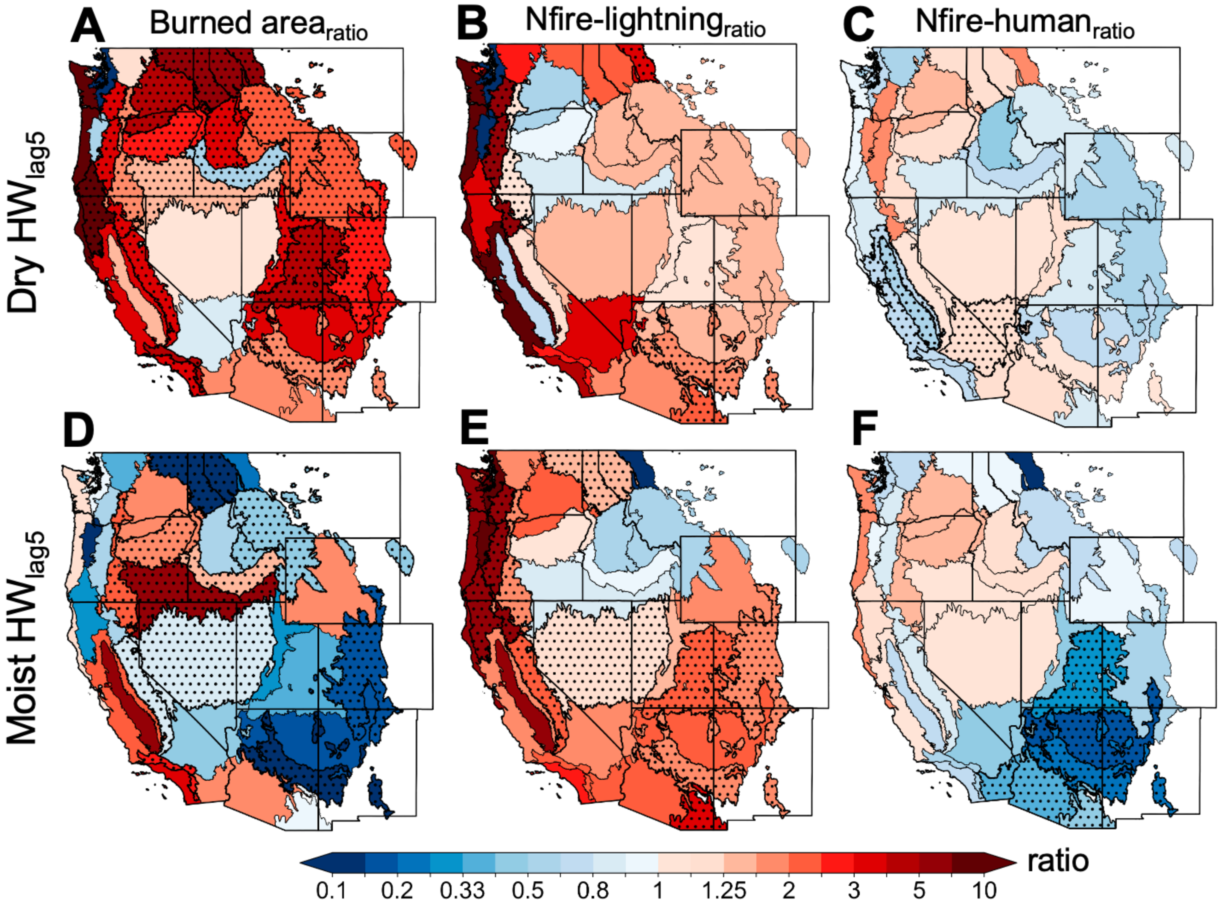

Fig. S10. As in Fig. 5, but for HW$_{lag5}$: comparing 1-5 days after the end of (A-C) dry heatwaves (HW) and (D-F) moist HW to the 5 days preceding both types of HW.

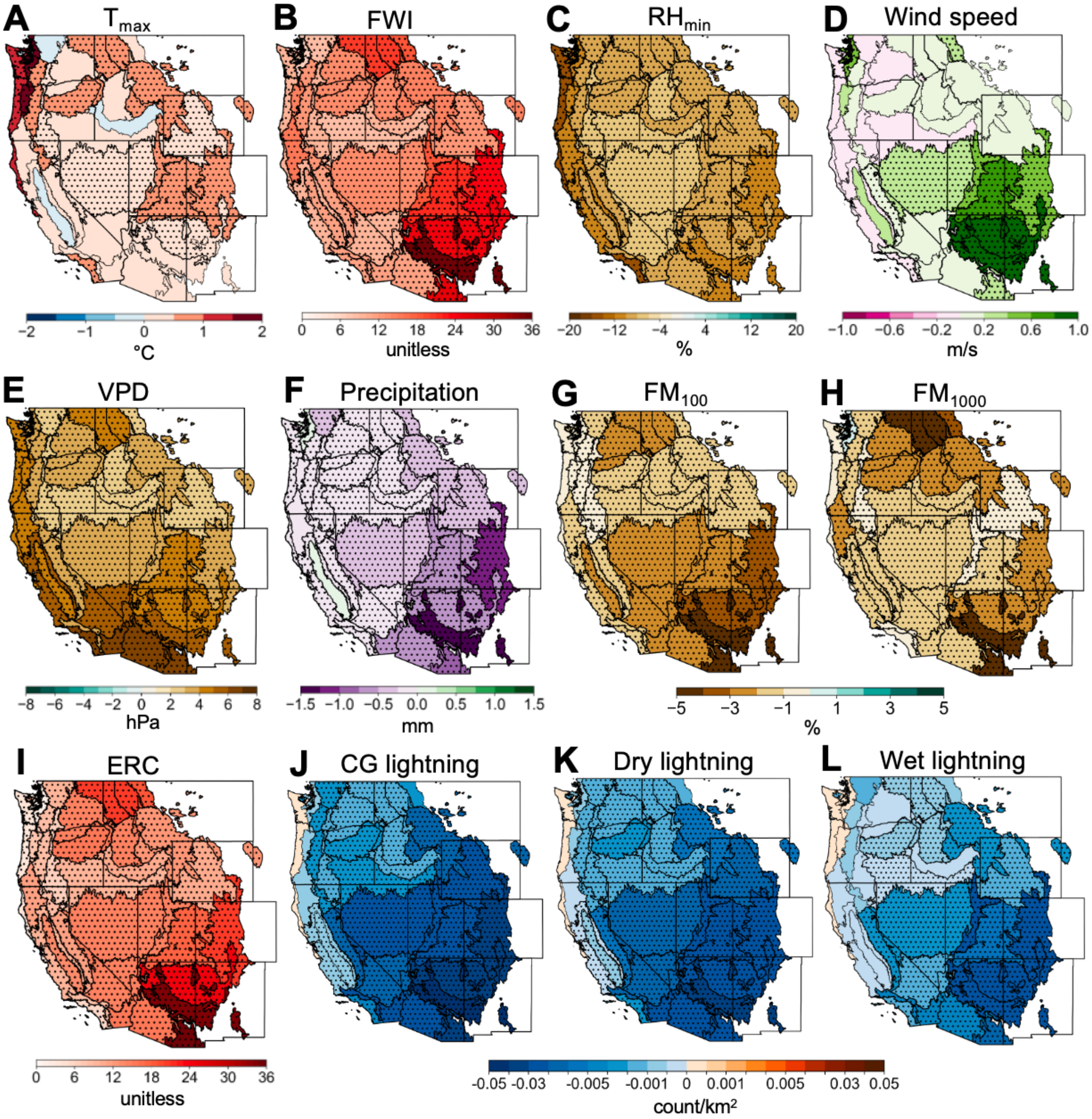

Fig. S11. Differences in meteorological and fuel moisture variables between dry and moist heatwaves (dry heatwaves minus moist heatwaves).

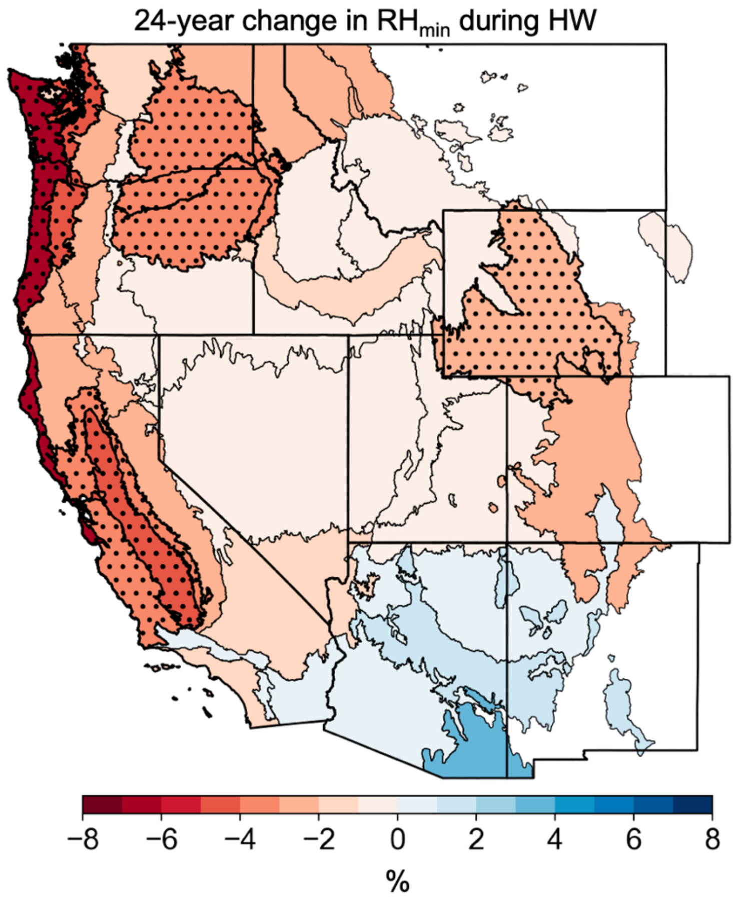

Fig. S12. As in Fig. 5I, but showing RH_{min} linear trends calculated by ecoregion and multiplied by the number of years (n = 24). Stippling indicates significant trends ($p < 0.10$) according to a two-tailed t-test.

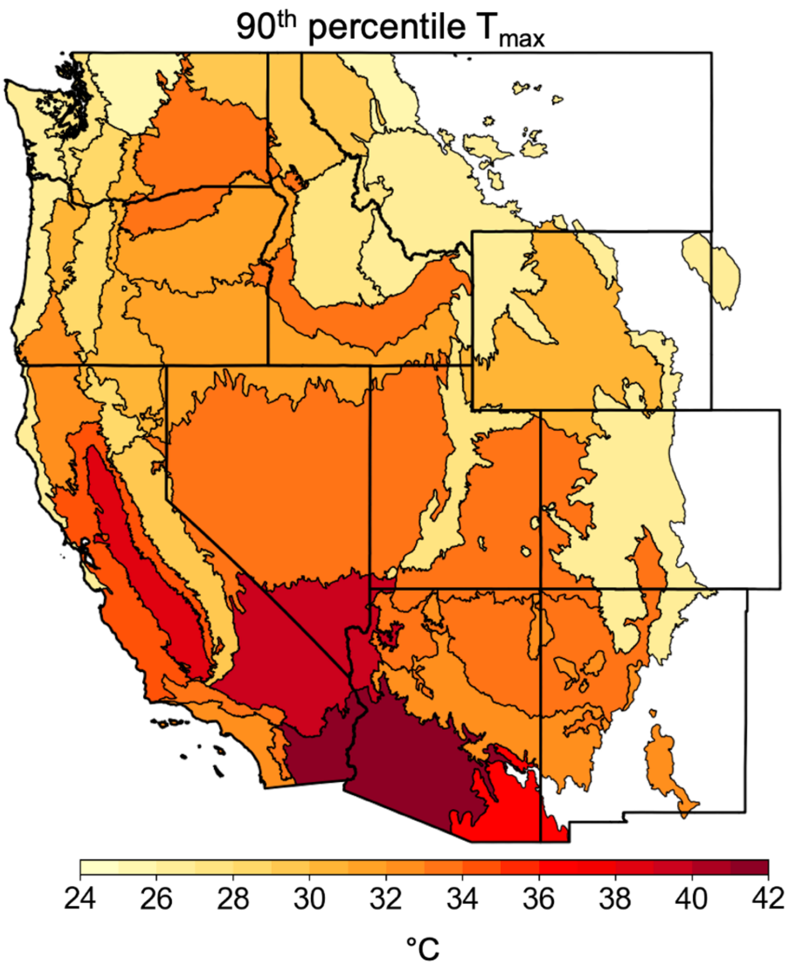

Fig. S13. T_{max} values corresponding to the 90th percentile of the warm-season distribution.
